# Identification of Novel HIV 1- Protease Inhibitors: Application of Ligand and Structure Based Pharmacophore Mapping and Virtual Screening

**DOI:** 10.1371/journal.pone.0048942

**Published:** 2012-11-08

**Authors:** Divya Yadav, Sarvesh Paliwal, Rakesh Yadav, Mahima Pal, Anubhuti Pandey

**Affiliations:** Department of Pharmacy, Banasthali University, Banasthali, Rajasthan, India; Concordia University Wisconsin, United States of America

## Abstract

A combined ligand and structure-based drug design approach provides a synergistic advantage over either methods performed individually. Present work bestows a good assembly of ligand and structure-based pharmacophore generation concept. Ligand-oriented study was accomplished by employing the HypoGen module of Catalyst in which we have translated the experimental findings into 3-D pharmacophore models by identifying key features (four point pharmacophore) necessary for interaction of the inhibitors with the active site of HIV-1 protease enzyme using a training set of 33 compounds belonging to the cyclic cyanoguanidines and cyclic urea derivatives. The most predictive pharmacophore model (hypothesis 1), consisting of four features, namely, two hydrogen bond acceptors and two hydrophobic, showed a correlation (*r*) of 0.90 and a root mean square of 0.71 and cost difference of 56.59 bits between null cost and fixed cost. The model was validated using CatScramble technique, internal and external test set prediction. In the second phase of our study, a structure-based five feature pharmacophore hypothesis was generated which signifies the importance of hydrogen bond donor, hydrogen bond acceptors and hydrophobic interaction between the HIV-1 protease enzyme and its inhibitors. This work has taken a significant step towards the full integration of ligand and structure-based drug design methodologies as pharmacophoric features retrieved from structure-based strategy complemented the features from ligand-based study hence proving the accuracy of the developed models. The ligand-based pharmacophore model was used in virtual screening of Maybridge and NCI compound database resulting in the identification of four structurally diverse druggable compounds with nM activities.

## Introduction

The pandemic spread of human immunodeficiency virus-1 (HIV-1), the etiologic agent of AIDS, has promoted an unending scientific effort to understand and control this disease. The resultant understanding of HIV-1 life cycle has defined many different targets for potential drug intervention. New recommendations of the International Antiviral Society- USA (IAS-USA) point towards the health benefits of early antiretroviral treatment (ART) [1]. The virally encoded homodimeric aspartyl protease (HIV Pr) enzyme is currently one of the most promising therapeutic targets for the treatment of AIDS due to its critical role in the virus maturation and replication. Protease-mediated maturation of HIV-1 virus particles is essential for virus infectivity [2]. The HIV-1 protease enzyme has a homodimeric C-2 symmetric structure and each monomer contributes one catalytic aspartic residue and flexible flap, which is able to bind the substrates and inhibitors [3]. In addition, a characteristic bound water molecule forms an hydrogen bonding network between the flaps and bond substrates creating a tetrahedral transition-state intermediate. These drugs target HIV-protease enzyme which is a proteolytic enzyme responsible for cleaving large polyprotein precursor into biologically active protein products. HIV polyprotein precursor is encoded by the gag and *gag-pol* genes. These genes encode the precursor with HIV structural core proteins and various viral enzymes, including the reverse transcriptase, the integrase, the RNAse H and the protease. The *pol* gene of the human immunodeficiency virus type 1 (HIV-1) encodes for the aspartic protease which mediates proteolytic processing of the *gag* and the *gag-pol* viral gene products liberating functional enzymes and structural proteins which are essential for the formation of the mature, infectious virus. The entire processing of *gag* and *gag-pol* precursors is finely coordinated and regulated by the activity of retroviral protease [4], [5]. Inactivation of the aspartic protease leads to the formation of noninfectious virions. Protease inhibitors represent a valid option in first line therapy of HIV-infected patients [6] and even their monotherapy has been shown to be effective in maintaining long-term viral suppression in a majority of patients [7]. Recently, many different classes of HIV-1 protease inhibitors have been developed, showing excellent antiviral profiles [8]–[13].

Two different approaches have been taken in the design of protease inhibitors, one involving targets which are peptidic in nature and another one employs non-peptidal character. However, peptidal protease inhibitors have shown low bioavailability and poor pharmacokinetics and normally possess multiple stereocentres [14]. Some have also reported artherogenic dyslipidemia [15] peripheral lipodystropy [16]. Hence, efforts have increasingly focused upon identifying non-peptidic HIV-1 protease inhibitors. Currently, licensed non-peptidal protease inhibitors include indinavir, ritonavir, saquinavir, and neflinavir. Some newer inhibitors with nonpeptide structure have also been developed, such as lopinavir, the cyclic urea mozinavir, atazanavir, tipranavir and the C2-symmetric protease inhibitor L-mannaric acid. In spite of having such a diversity of drugs available for treatment of HIV infections, millions of dollars are being spent on AIDS research for developing new drugs. Drug-related side effects, toxicity, and the development of drug-resistant HIV strains is a compelling reason for more efforts to develop newer inhibitors [17]. Resistance arises from mutations in the viral genome, specifically in the regions that encode the molecular targets of therapy, i.e. HIV-1 protease enzymes. These mutations alter the viral enzymes in such a way that the drug no longer inhibits the enzyme functions and the virus restores its free replication power. Moreover, the rate at which the virus reproduces and the high number of errors made in the viral replication process creates a large amount of mutated viral strains [18]. Thus, resistance toward the marketed HIV-1 protease inhibitors is a serious threat to efficient HIV treatment. Moreover, many of the HIV-1 protease inhibitors in the market suffer from poor pharmacokinetic properties due to poor aqueous solubility, low metabolic stability, high protein binding, and poor membrane permeability. The development of new HIV-1 protease inhibitors addressing these issues is therefore of high importance. Hence, a computational analysis that includes ligand and target based drug design approach has been used to identify new lead compounds with high potency.

A pharmacophore represents the 3D arrangements of structural or chemical features of a drug (small organic compounds, peptides, peptidomimetics, etc.) that may be essential for interaction with the target/optimum binding. These pharmacophores can be used in different ways in drug design programs: (1) as a 3D query tool in virtual screening to identify potential new compounds from 3D databases of “drug-like” molecules with patentable structures different from those already discovered; (2) to predict the activities of a set of new compounds yet to be synthesized; (3) to understand the possible mechanism of action [19], [20].

The aim of the reported endeavor was to generate pharmacophore models for HIV-1 protease inhibitors through analog-based pharmacophore generation process (HypoGen algorithm) which employed a set of cyclic cyanoguanidines and cyclic urea ligands that have been experimentally observed to interact with a HIV-1 protease enzyme and also to compare these models with those obtained in a structure-based approach to identify novel structural characteristics and scaffolds for HIV-1 protease.

The aspired aim was achieved by development of validated, robust and highly predictive pharmacophore models from both ligand and structure based approaches. The validity of the pharmacophore models was established by Fischer’s randomization test, internal and external test set predictions. The complementary nature of ligand and structure-based model has augmented the statistical findings of both the pharmacophores. The significance of the present study is clearly reflected by the identification of four highly potent lead compounds as protease inhibitors.

## Materials and Methods

### Ligand Based 3D Pharmacophore Generation

All molecular modeling calculations were performed on recent software package Catalyst [21] which has an in-build pharmacophore generation facility. Catalyst is an integrated commercially available software package that generates pharmacophores, commonly referred to as hypotheses. It enables the use of structure and activity data for a set of lead compounds to create a hypothesis, thus characterizing the activity of the lead set [22]. HypoGen algorithm in Catalyst allows identification of hypotheses that are common to the “active” molecules in the training set but at the same time not present in the “inactives” [23].

**Figure 1:**
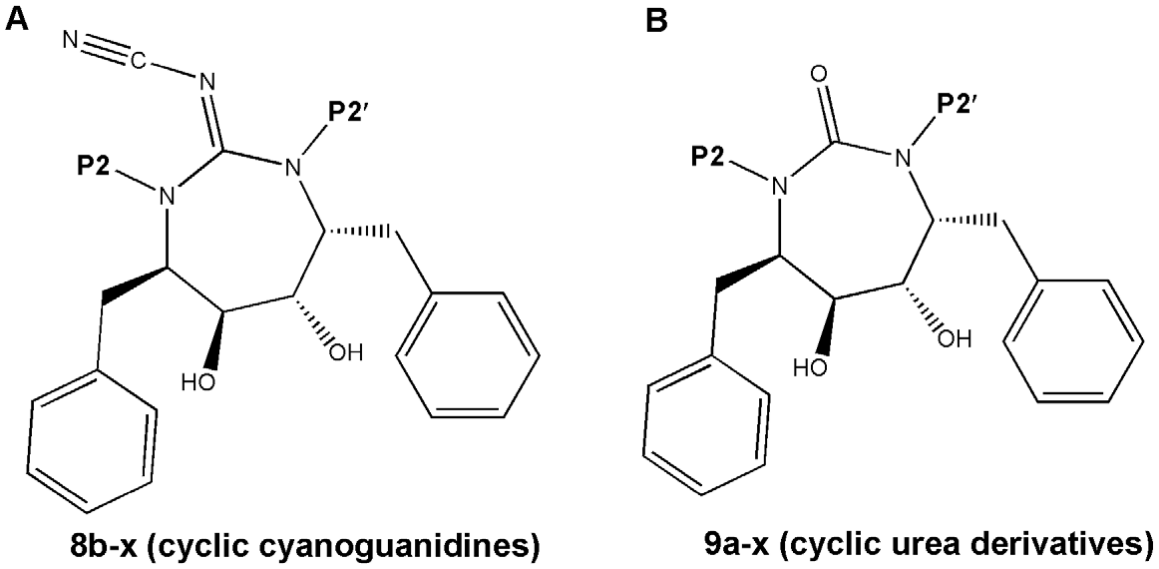
Chemical structures of protease inhibitors. (A) Cyclic cyanoguanides. (B) Cyclic urea derivatives.

A series of 47 compounds belonging to the cyclic cyanoguanidines and cyclic urea derivatives and their corresponding biological data represented as Ki values in nM reported by Jadhav et al. [24] (structures reported in Figure 1 and Table 1) were employed for the present pharmacophore generation study in view of the following reasons: (1) pharmacophore modeling studies have not been performed on this series, (2) series under consideration exhibit well defined biological activities of its compounds, (3) the compound in the series has large variation in biological activity for small change in the structure, (4) maximum variation in the biological activity (i.e. their order of magnitude was more than 4), and (5) diversity in the structures [25]. All the molecules under consideration were randomly split into training and test set. Training and test set were comprised of 33 and 14 compounds respectively. Energy minimization was carried using CHARMM force field. The Catalyst software reconfigure the generated structures at the minimum potential energy form using CHARMM force field. The CHARMM program in Catalyst allows generation and analysis of a wide range of molecular simulations [26].

**Table 1:** Cyclic cyanoguanides and cyclic urea derivatives with various substitutions at P2/P2′ positions and their biological activity data utilized for present work.

Compound name	P2/P2'	Biological Activity Ki (nM) values
8b	allyl	37
8c	*n*-propyl	14
8d	*n*-butyl	2.7
8e	3,3-dimethylallyl	30
8f	3-methylbutyl	3.8
8g	cyclopropylmethyl	22
8h	cyclobutylmethyl	2
8i	cyclopentylmethyl	1.5
8j	cyclohexylmethyl	5.7
8k	benzyl	20
8l	3-nitrobenzyl	89
8m	4-nitrobenzyl	67
8n	3-aminobenzyl	7.4
8o	4-aminobenzyl	25
8p	3-cyanobenzyl	27
8q	4-cyanobenzyl	128
8r	3-hydroxybenzyl	0.72
8s	4-hydroxybenzyl	2.6
8t	3-(benzyloxy)benzyl	1370
8u	4-(benzyloxy)benzyl	900
8v	3-(hydroxymethyl)benzyl	1.7
8w	4-(hydroxymethyl)benzyl	11
8x	2-naphthylmethyl	22
9a	H	267
9b	allyl	5.2
9c	*n*-propyl	8
9d	*n*-butyl	1.4
9e	3,3-dimethylallyl	1.6
9f	3-methylbutyl	12
9g	cyclopropylmethyl	2.1
9h	cyclobutylmethyl	1.3
9i	cyclopentylmethyl	4.3
9j	cyclohexylmethyl	37
9k	benzyl	3
9l	3-nitrobenzyl	2.8
9m	4-nitrobenzyl	32
9n	3-aminobenzyl	0.28
9o	4-aminobenzyl	1.1
9p	3-cyanobenzyl	3
9q	4-cyanobenzyl	52
9r	3-hydroxybenzyl	0.12
9s	4-hydroxybenzyl	0.12
9t	3-(benzyloxy)benzyl	340
9u	4-(benzyloxy)benzyl	542
9v	3-(hydroxymethyl)benzyl	0.14
9w	4-(hydroxymethyl)benzyl	0.34
9x	2-naphthylmethyl	0.31

The Catalyst model treats the molecular structures as templates comprising chemical functions localized in space that will bind effectively with complementary functions on the respective binding proteins. The most relevant chemical features are extracted from a small set of compounds that cover a broad range of activity. Molecular flexibility is taken into account by considering each compound as an ensemble of conformers representing different accessible areas in 3D space. The conformation is of great importance for the mode of drug action since it relies on the easy accessibility of the reactive groups. Conformations for all molecules under study were generated using the “best” option (the program has the ability to modify the conformations of molecules during execution to provide a more precise database/spreadsheet search; the best algorithm finds the best fit among conformations, permitting no conformer’s energy to rise by more than the default value) with an energy cut-off of 20 kcal/mol. The maximum number of conformations to be generated for any molecule was set to 250. This is because Catalyst considers only the first 250 conformations in hypothesis generation [25]. Catalyst generates random conformations (using a “polling” algorithm) to maximally span the accessible conformational space of a molecule and not necessarily only the local minima. In this light, the conformational models of the compounds will include some higher-energy structures that may be meaningful for receptor binding, since potentially favorable interactions (e.g., hydrogen bonding) with the latter will then compensate for the excessive conformational energy [27].

**Figure 2:**
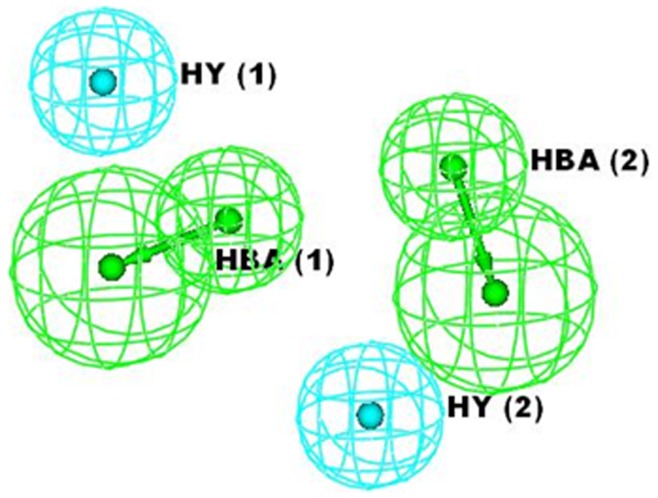
Pharmacophoric features identified from best hypothesis 1.

### Generation of Pharmacophores

All molecules in the training set along with their conformations were used for hypothesis (pharmacophore) generation within Catalyst, which aims to identify the best 3-dimensional arrangement of chemical functions explaining the activity variations among the compounds in the training set. HypoGen tries to find hypotheses that are common among the active compounds of the training set but do not reflect the inactive ones [28]. Instead of using just the lowest energy conformation of each compound, all the conformational models for molecules in each training set were used for pharmacophore hypothesis generation. During the hypothesis generation exercise, it was observed that four features, i.e., two hydrogen bond acceptor-lipid (HBA) and two hydrophobic (HY) features, dominated in most of the useful hypotheses generated by the Catalyst software. Therefore, these four features were used to generate 10 pharmacophore hypotheses with top ranking scores from the training set, using a default uncertainty value Δ (an uncertainty Δ value in the Catalyst paradigm indicates an activity value lying somewhere in the interval from “activity divided by Δ” to “activity multiplied by Δ”) of 3 and MinPoints and MinSubsetPoints values of 4 (default value). The MinPoints parameter controls the minimum number of location constraints required for any hypothesis. The MinSubsetPoint parameter defines the number of chemical features that a hypothesis must match in all the compounds set [29]. HypoGen process returned ten pharmacophore models with top ranking scores. The quality of the generated pharmacophore models was evaluated using a cost function analysis, Fisher’s randomization test, internal and external test set prediction.

**Figure 3:**
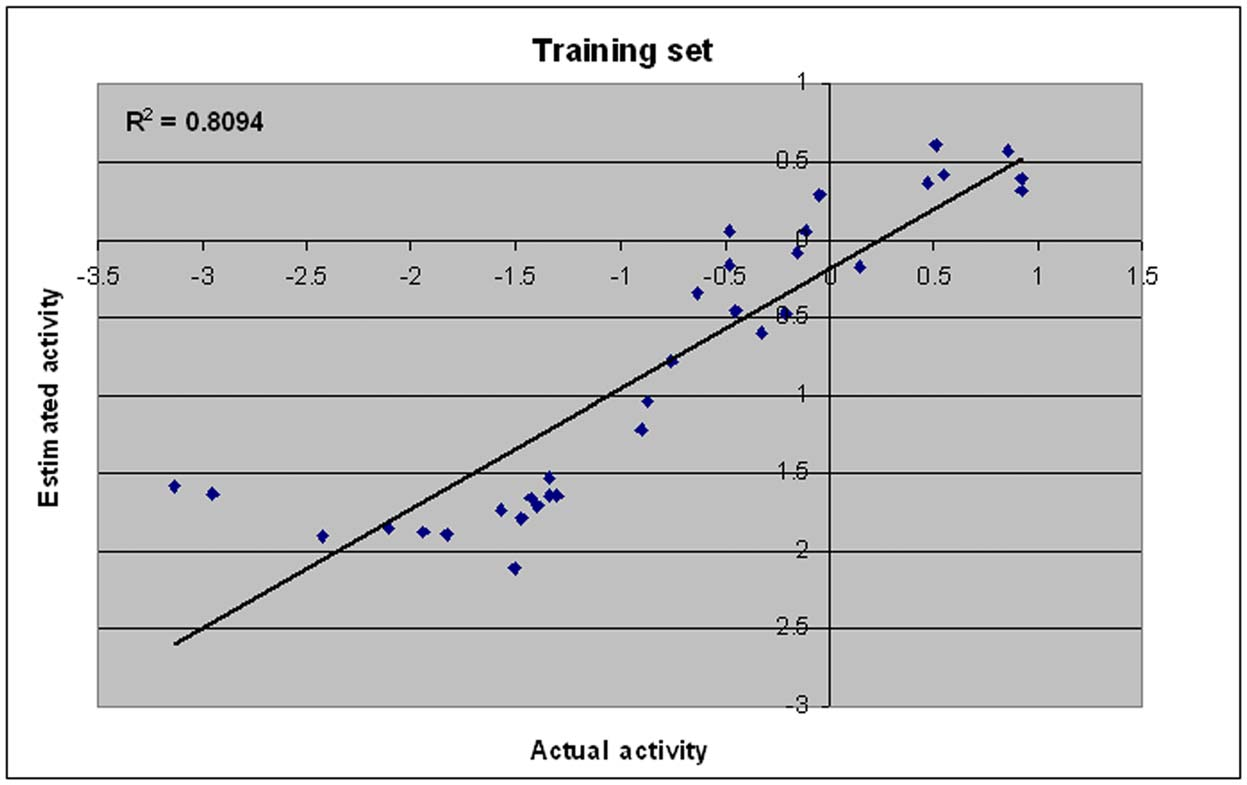
A plot of actual versus estimated biological activity for training set compounds.

### Evaluation of the HypoGen Model

#### 1. Cost function analysis

The evaluation of the quality of the generated pharmacophoric hypothesis was carried out on the basis of cost value (total cost) which consists of three components namely, the weight cost, the error cost and the configuration cost. The weight component increases in a Gaussian form as the feature weight deviates from the idealized value of 2.0. The error cost increases as the RMS distance between the estimated and the measured activities for the training set increases. The configuration cost represents the complexity or the entropy of the hypothesis space being optimized and is constant for a given data set. It depends on the complexity of the pharmacophore hypothesis space. Any value higher than 17 may indicate that the correlation from any of the generated hypothesis is most likely due to chance, so either some attention has to be given in the selection of training set molecules or the entropy cost should be reduced by limiting the minimum and maximum features. Generated pharmacophore was also tested by another two important cost calculations i.e. fixed cost and the null cost, the former represents the simplest model that perfectly fits the data and the latter i.e. null cost is the cost of a pharmacophore without any feature where the calculated activity data of each molecule in the training set is the average value of all activities. In Catalyst software the differences between the cost of the generated and the null hypothesis should be as large as possible; a value of 40–60 bits difference may indicate that there is only 75–90% chance of representing a true correlation in the data set used. The total cost of any hypothesis should be nearer to the value of the fixed cost for any meaningful model. The rms deviation represents the quality of the correlations between the estimated and the actual data [30].

**Table 2:** Performance of top ten pharmacophoric hypotheses generated.

Hypo. No.	Total cost	Correlation (r)	RMS	Weight	Configuration	Features	r^2^ (training)	r^2^ (test)
1	143.96	0.90	0.71	1.96	15.42	2 HBA, 2 HY	0.80	0.77
2	150.34	0.85	0.83	1.97	15.42	2 HBA, 2 HY	0.79	0.76
3	153.88	0.82	0.86	1.99	15.42	2 HBA, 2 HY	0.79	0.75
4	155.55	0.81	0.87	1.93	15.42	2 HBA, 2 HY	0.75	0.73
5	156.22	0.81	0.89	2.47	15.42	2 HBA, 2 HY	0.77	0.64
6	156.64	0.82	0.88	2.64	15.42	2 HBA, 2 HY	0.76	0.13
7	156.74	0.80	0.98	1.03	15.42	2 HBA, 2 HY	0.71	0.45
8	157.24	0.80	0.99	1.99	15.42	2 HBA, 2 HY	0.72	0.46
9	157.27	0.79	1.01	1.96	15.42	2 HBA, 2 HY	0.74	0.14
10	157.31	0.80	1.03	2.79	15.42	2 HBA, 2 HY	0.76	0.64

#### 2. CatScramble validation

An additional validation technique, known as CatScramble which is based on Fischer’s randomization test was applied. In this test, the biological data and the corresponding structures are scrambled several times and the software is challenged to generate pharmacophoric models from the randomized data. The confidence in the parent hypotheses (i.e., generated from unscrambled data) is lowered proportional to the number of times the software succeeds in generating binding hypotheses from scrambled data of apparently better cost criteria than the parent hypotheses. The statistical significance is given by the equation.
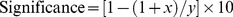
(1)


**Table 3:** Pharmacophoric features and corresponding weights, tolerances, and 3D coordinates of successful model.

Chemical Features								
Model	definitions		HBA Lipid (1)		HBA Lipid (2)		HY (1)	HY (2)
Hypothesis 1	weights		2.34		2.34		2.34	2.34
	tolerances		1.60	2.20	1.60	2.20	1.60	1.60
	coordinates	X	–1.24	–3.37	2.89	3.62	1.18	–4.22
		Y	0.24	–0.19	0.39	–2.54	–4.76	2.84
		Z	2.72	4.84	–0.76	–0.95	0.88	1.40

where *x* = total number of hypotheses having a total cost lower than best significant hypothesis and *y* = number (HypoGen runs initial+random runs). To obtain a 95% confidence level, 19 random spreadsheets are generated (*y* = 20) and every generated spreadsheet is submitted to HypoGen using the same experimental conditions (functions and parameters) as the initial run [31]. The pharmacophore hypothesis generated for present HIV-1 protease inhibitors included in the training set were evaluated for their statistical significance using the aforesaid CatScramble program.

#### 3. Internal test set prediction

The ability of the models to predict the biological activity of compounds outside the model development procedure is a common method of validation. An internal test set comprising of 14 compounds was employed to assess statistical significance of the developed model. Here in this case test set prediction was measured in terms of squared correlation coefficient (*r^2^*). All the selected derivatives were mapped onto the generated pharmacophoric model and thus prediction of the desired activity was made. The best mapped compound was estimated and compared to those with least mapped features. The Catalyst program fits each compound to a hypothesis and reports back a series of ‘Fit’ scores. The fit function does not depend only on the mapping of the feature but also possess a distance term measuring the distance between the feature on the molecule and the centroid of the hypothesis feature, and both these terms are used in the calculation of geometric fitness [32]. A relationship between log (activities) and the corresponding fit-values for all test set molecules was computed using linear regression after mapping of each molecule to the hypothesis.

#### 4. External test set validation

In order to access the predictive power of the resulting HypoGen pharmacophoric model, an external test set comprising of similar (cyclic urea analogs) and different (market drugs which were non cyclic ureas) structural types was used to validate the four-feature pharmacophore. Out of fifteen molecules from external test set, first five molecules are market drugs (saquinavir, indinavir, nelfinavir, ritonavir and 141W94 ) having diverse structure (non cyclic ureas), while another ten molecules were cyclic urea analogs which were selected on the basis of two most active molecules from five different published literature. Ki values for all external test set candidates have been determined in the same laboratory as that of training and internal test set compounds, using comparable biological assays. These fifteen external test set molecules were mapped onto the HypoGen pharmacophore and their mapping fashion were analyzed and the pharmacophore also predicted their biological activities which were compared with their actual activities.

### Structure Based 3D Pharmacophore Generation

When the three-dimensional (3D) structure of the enzyme/target is available, structure-based pharmacophore techniques can also be applied to improve the drug design process. In this study, a structure-based pharmacophore identification approach was employed to augment the findings of ligand based pharmacophore.

### Methodology

The three-dimensional structure of HIV-1 protease enzyme complexed with inhibitor *L-700,417* was used to develop a pharmacophore model. 47 compounds from same series of HIV-1 protease inhibitor analogs belonging to the cyclic cyanoguanidines and cyclic urea derivatives which were employed for ligand based study were used as a validation set for mapping onto the developed pharmacophore to uncover the putative binding site and structural requirement of the protease inhibitors.

### Defining Active Site and Interaction Generation

X-ray crystal structure of the HIV-1 protease complex (obtained from protein data bank with PDB entry 4PHV) with inhibitor named *L-700,417,* which is a HIV-1 protease inhibitor with Pseudo *C2* Symmetry, was used for structure based pharmacophore generation [33]. The protein structure was monitored for valence and the missing hydrogen were added, the structure was further checked using protein health check tool for any structural error. The cleaned enzyme structure was subjected to active site identification. The receptor active site was identified using a sphere whose location and radius was adjusted to 9.0 Å, so as to include the active site and the key residues of the protein involved in interaction with ligands. Keeping the density of lipophilic sites and density of polar sites parameter value to 10, the interaction map was generated [34].

### Creating Pharmacophore Model Based on the Interactions and Searching Compound Library

The interaction map often displays a large number of features, especially when the receptor is capable of binding a variety of ligands and has a number of different binding modes. Thus, deriving pharmacophore models directly from the interaction map can be quite complicated. To overcome this problem, neighboring features of the same type were grouped to the same cluster. The feature closest to the geometric center of the cluster was selected to represent the cluster, whereas the rest of the features were omitted. However, even after clustering the numbers of the features were still too high to use all of them in a single query. A query composed of all the features may fail to retrieve any hits from the database/compound library. Therefore, multiple 3D queries, composed of fewer numbers of features, were generated from the interaction map by considering all the possible combinations. The final model constructed was subjected to non feature atoms exclusion. The exclusion constraint feature is an object that represents an excluded volume in space, within a given radius. The excluded volumes were placed on regions of space that are occupied by the inactive molecules but not the active molecules. A pharmacophore with an excluded volume only matches if no atoms penetrate the excluded area [35]. The final hypothesis contained five features: one hydrogen bond donors and two hydrogen bond acceptors and two hydrophobic groups (with additional 10 excluded volumes) describing the interactions between the protein HIV-1 protease and the ligand *L-700,417*.

In order to validate the hypothesis, different conformations for 47 HIV-1 protease inhibitor analogs belonging to the cyclic cyanoguanidines and cyclic ureas were used as validation data set. All the compounds and their conformations were mapped onto the developed five-feature pharmacophore. Moreover, 15 external test set molecules which were used to validate the pharmacophore developed from ligand-based methodology were also used as a validation set and were screened on the five-feature structure-based pharmacophore and their mapping fashion were analyzed.

### Database Screening

Catalyst-generated best pharmacophore model comprising of best selected chemical features were used as query for searching the chemical 3D databases (Maybridge and NCI) [36]. Virtual screening of such databases can serve two main purposes: first, validating the quality of the generated pharmacophore models by selective detection of compounds with known inhibitory activity, and, second, finding novel, potential leads suitable for further development [37]. Thus, with the purpose of identifying novel lead compounds, the four-feature pharmacophore model obtained from HypoGen analysis was used as a three-dimensional query for database search. As a result of this search, 399 lead compounds were obtained from the 3D query and their activities were estimated, out of which 4 candidates emerged as potential ligands exhibiting a good perfect four feature fit. To explore the druggability of the molecules, ADME (absorption, distribution, metabolism, and excretion) properties were checked by applying Lipinski’s rule on all the four compounds obtained from database screening. Violation in number of HBD (hydrogen bond donor), HBA (hydrogen bond acceptor), molecular weight, and LogP were detected [38].

As an additional validation setup, all the four identified lead compounds were mapped onto the structure-based pharmacophore. The mapping pattern was observed to augment the confidence in identified novel lead structures.

## Results and Discussion

### Ligand Based 3D Pharmacophore Generation

The HypoGen algorithm of Catalyst applied on the training set of 33 compounds with anti HIV-1 protease inhibitory activity (Table 1) resulted in the generation of 10 pharmacophore hypothesis. The quality of the generated pharmacophore hypotheses was evaluated by considering the cost functions represented in bits unit calculated by HypoGen module during pharmacophore generation. The fixed cost of the 10 top-scored hypotheses was 137.4 bits, well separated from the null hypothesis cost of 200.49 bits. The cost values, correlation coefficients (r), RMSD, and features for the top ten hypotheses are listed in Table 2. The total hypothesis cost, expressed in bits, of the 10 best hypotheses varies from 143.9 to 157.3. Such a range, covering only 14 bits, suggests that the set of the generated hypothesis is homogeneous and that the selected training set is adequate for pharmacophore design. From the table we can see all the 10 hypotheses including the best hypothesis 1 have the same four features, viz., two hydrogen bond acceptor lipid (HBA) and two hydrophobic (HY) features. Pharmacophore features, ranking scores, and statistical parameters associated with the generated hypotheses are listed in Table 3. The top-ranked pharmacophore model (hypothesis 1) was marked by best predictive power and statistical significance as described by the high correlation coefficient of *r* = 0.90, r^2^ = 0.81, low root mean-square deviation, rmsd = 0.71, weight = 1.96, error cost = 126.58 and cost difference = 56.59, satisfying the acceptable range recommended in the cost analysis of the Catalyst procedure [39]. The configuration cost was 15.42, indicating that all generated models have been thoroughly analyzed. In the standard HypoGen mode, the configuration cost should not exceed a maximum value of 17 (corresponds to a number of 2**^17^** pharmacophore models) because high values may lead to chance correlation of the generated hypothesis, since Catalyst cannot consider more than 2**^17^** models in the optimization phase, and so the rest are left out of the process. The cost difference between total and fixed costs for the best hypothesis was only 6.5 bits, indicating the high probability of the true correlation of the data. Lower the cost difference between the total and fixed costs, the higher the probability is for the true correlation of the data. Thus, hypothesis 1 was retained for further analysis as the best pharmacophore model for HIV-1 protease inhibitory activity with four features, viz., two hydrogen bond acceptor lipid (HBA) and two hydrophobic (HY) features, is statistically the most relevant model (Fig. 2). Green and blue color is represented by HBA and HY features respectively. Once hypothesis 1 was identified as the best-ranked model, it was subjected to further evaluation for its predictive ability. The hypothesis 1 model was utilized to predict the activities of all 33 training compounds. Hypothesis 1 has estimated the activity of the training set molecules accurately. In this study all compounds were classified by their activity as highly active (<5 nM, +++), moderately active (5–70 nM, ++) and inactive (>80 nM, +). The scored estimated activities of training set using hypothesis 1 along with their corresponding error values are shown in Table 4. A plot between the observed versus estimated activity demonstrated a good correlation coefficient (*r* ^2^ training = 0.80) for training set molecules within the range of uncertainty 3, indicating the high predictive ability of the pharmacophore (Fig. 3).

**Table 4:** Actual and estimated Ki (nM) values of training set molecules based on model hypothesis 1.

Name	Actual Ki(nM) values	Estimated Ki (nM) values	Activity scale (Actual)	Activity scale (Estimated)	Error	Fit value	Mapped Feature			
							HBA 1	HBA 2	HY 1	HY 2
9r	0.12	0.404	**+++**	**+++**	3.3	8.86	1	1	1	1
9s	0.12	0.485	**+++**	**+++**	3.9	8.79	1	1	1	1
9v	0.14	0.27	**+++**	**+++**	2	9.04	1	1	1	1
9n	0.28	0.379	**+++**	**+++**	1.4	8.89	1	1	1	1
9x	0.31	0.244	**+++**	**+++**	1.3	9.09	1	1	1	1
9w	0.34	0.429	**+++**	**+++**	1.2	8.84	1	1	1	1
8r	0.72	1.495	**+++**	**+++**	2.1	8.29	1	1	1	1
9o	1.1	0.505	**+++**	**+++**	2.3	8.77	1	1	1	1
9h	1.3	0.863	**+++**	**+++**	1.5	8.54	1	1	1	1
9d	1.4	1.231	**+++**	**+++**	1.1	8.38	1	1	1	1
9e	1.6	2.916	**+++**	**+++**	2.1	7.99	1	1	1	1
9g	2.1	3.981	**+++**	**+++**	1.6	7.88	1	1	1	1
9l	2.8	2.886	**+++**	**+++**	1	8.01	1	1	1	1
9k	3	1.452	**+++**	**+++**	2.1	8.31	1	1	1	1
9p	3	0.881	**+++**	**+++**	3.2	8.53	1	1	1	1
9i	4.3	2.214	**+++**	**+++**	1.8	8.12	1	1	1	1
8j	5.7	5.987	**++**	**++**	1.1	7.70	1	1	1	1
8n	7.4	10.84	**++**	**++**	1.5	7.46	1	1	1	1
9c	8	16.455	**++**	**++**	2	7.25	1	1	1	1
8k	20	44.099	**++**	**++**	2.2	6.83	–	1	1	1
8g	22	33.971	**++**	**++**	1.5	6.94	–	1	1	1
8x	22	43.845	**++**	**++**	2	6.83	–	1	1	1
8o	25	51.857	**++**	**++**	2.1	6.75	–	1	1	1
8p	27	44.839	**++**	**++**	1.6	6.82	–	1	1	1
8e	30	60.96	**++**	**++**	2.1	6.68	–	1	1	1
9m	32	127.027	**++**	**+**	4	6.37	1	1	1	-
8b	37	54.725	**++**	**++**	1.5	6.78	–	1	1	1
8m	67	76.781	**++**	**++**	1.1	6. 83	–	1	1	1
8l	89	74.638	**+**	**++**	1.2	6.59	–	1	1	1
8q	128	70.787	**+**	**++**	1.8	6.67	–	1	1	1
9a	267	79.729	**+**	**++**	3.4	6.59	–	1	1	1
8u	900	43.025	**+**	**++**	21	6.87	–	1	1	1
8t	1370	37.937	**+**	**++**	35	6.89	–	1	1	1

### Model Validation

Following validation approaches were adopted and their results were checked to ensure the accuracy of the model.

#### 1. Internal test set validation

**Figure 4:**
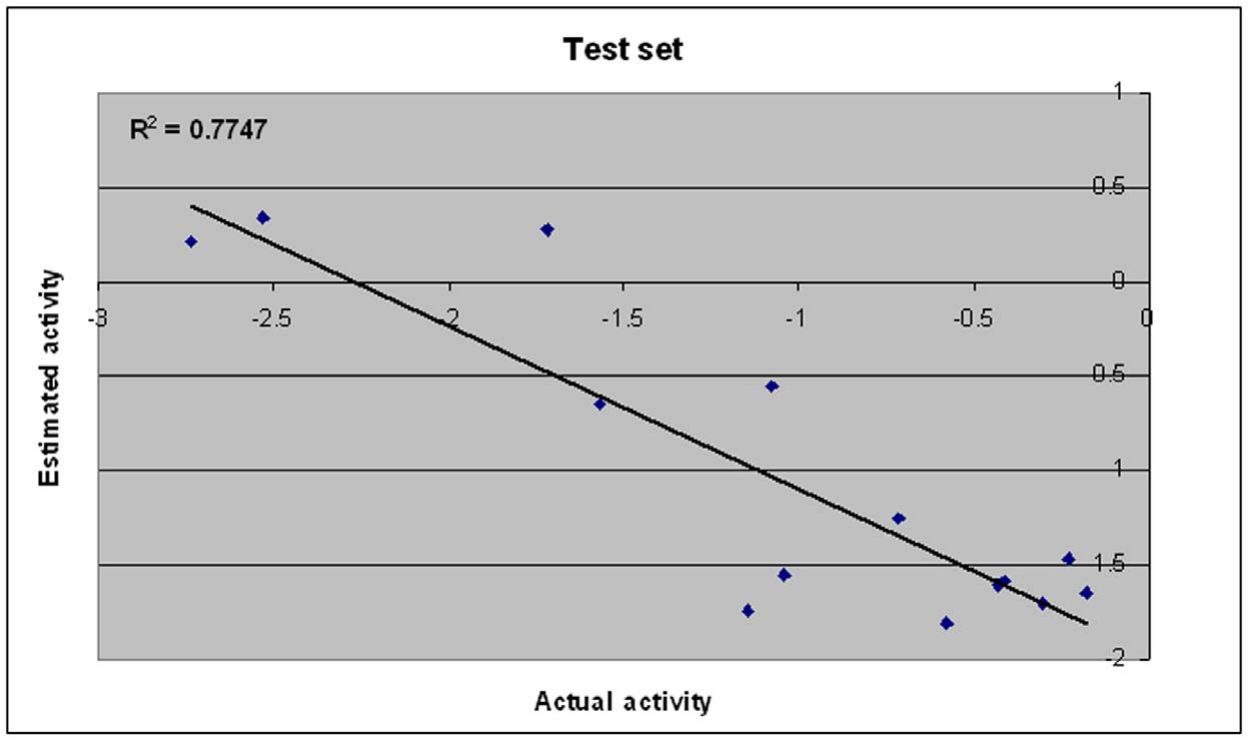
A plot of actual versus estimated biological activity for test set compounds.

The purpose of the pharmacophore hypothesis generation is not just to predict the activity of the training set compounds accurately but also to verify whether the pharmacophore models are capable of predicting the activities of compounds not included in the training set. A test set consisting of 14 ligands was subjected to phramcophore mapping analysis using the developed model. Objective of test set prediction was to verify whether generated pharmacophore models are capable of predicting the activities and classifying them correctly as actives or inactives. All molecules in the test set were built, minimized and subjected to conformational analysis like the molecules in the training set. Finally the compounds were mapped onto the best hypothesis using the best fit and a conformational energy constraint of 10 kcal mol^−1^. The scored estimated activities of test set compounds using hypothesis 1 as the pharmacophore are shown in Table 5. A correlation coefficient of 0.77 generated using the test set compounds shown in Fig. 4 indicates a good correlation between the actual and estimated activities, which means the hypothesis 1 is convictive.

**Table 5:** Actual and estimated Ki (nM) values of test set molecules based on model hypothesis 1.

Name	Actual Ki (nM) values	Estimated Ki (nM) values	Activity scale (Actual)	Activity scale (Estimated)	Fit value	Mapped Feature			
						HBA 1	HBA 2	HY 1	HY 2
8i	1.5	44.102	**+++**	**++**	6.826	–	1	1	1
8v	1.7	29.233	**+++**	**++**	7.004	1	1	1	1
8h	2	50.647	**+++**	**++**	6.765	–	1	1	1
8s	2.6	38.188	**+++**	**+++**	6.888	–	1	1	1
8d	2.7	40.345	**+++**	**+++**	6.864	-	1	1	1
8f	3.8	63.964	**+++**	**+++**	6.664	-	1	1	1
9b	5.2	18.108	**++**	**++**	7.212	1	1	1	1
8w	11	35.758	**++**	**++**	6.917	-	1	1	1
9f	12	3.568	**++**	**++**	7.918	1	1	1	1
8c	14	55.755	**++**	**+++**	6.724	-	1	1	1
9j	37	4.463	**++**	**++**	7.82	1	1	1	1
9q	52	0.531	**++**	**++**	8.745	1	1	1	1
9t	340	0.457	**+**	**++**	8.81	1	1	1	1
9u	542	0.62	**+**	**++**	8.677	1	1	1	1

#### 2. CatScramble validation

**Figure 5:**
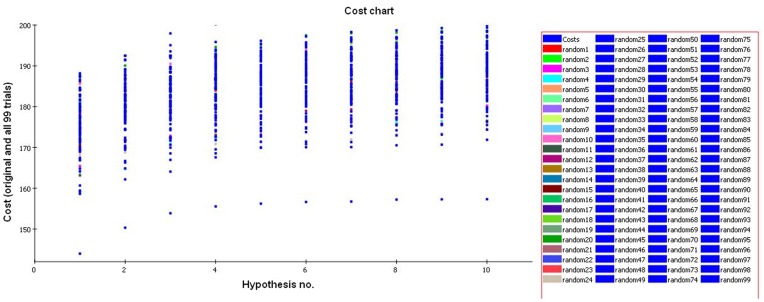
Graph of 99% catscrambled cost data. None of the outcome hypotheses had a lower cost score than the initial (best) hypothesis.

**Figure 6:**
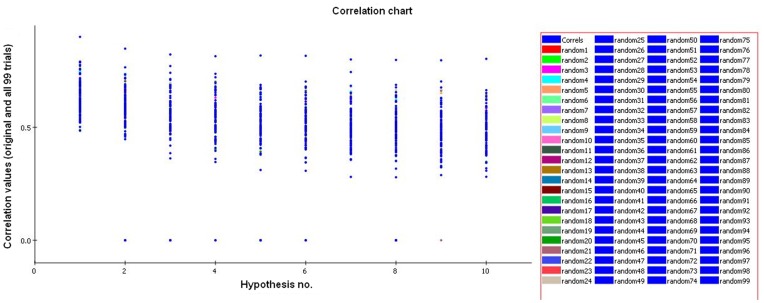
Graph of 99% catscrambled correlation data. None of the outcome hypotheses had a higher correlation score than the initial (best) hypothesis.

To further evaluate the statistical relevance of the model, the Fischer validation method at the confidence level of 99% was applied to the developed HypoGen model and thus 99 spreadsheets were generated. These random spreadsheets were used to generate hypotheses employing exactly the same features as used in generating the initial hypothesis. The experimental activities in the training set were scrambled randomly using CatScramble program, and the resulting training set was used for a HypoGen run. In this manner all parameters were taken from the initial HypoGen calculation. None of the outcome hypotheses had a lower cost score than the initial hypothesis (Fig. 5) which verifies that the hypothesis 1 was not obtained by chance. The data of cross validation clearly indicates that all values generated after randomization produced hypotheses with no significant value. Out of 99 runs, all trials had a correlation value less than 0.90 (Fig. 6), and also RMS deviation and total cost were very high, which is not desirable for a good hypothesis. Thus, validation method adopted provided strong confidence on the pharmacophore hypothesis 1.

#### 3. External test set validation

In order to finally validate our pharmacophore hypothesis, we used an external test set consisted of 15 molecules with Ki activity having similar and different structural information. The test set molecules were mapped onto the best pharmacophore hypothesis 1 and the actual activity versus estimated activity are shown in Table 6. All the external test set candidates exhibited a perfect four-feature mapping with good fit values. It would be very interesting to mention that estimated biological activities of market drugs such as saquinavir, indinavir, nelfinavir, ritonavir and 141W94 were very close to their corresponding actual activities. Hence, this proves the predictability of our developed pharmacophore.

**Table 6:** Table summarizing the results of external set molecules used as a validation tool.

S. No.	Name of external test set molecule	Actual Ki (nM) values	Estimated Ki (nM) values	Fit Value	Reference
1.	Saquinavir (non-cyclic ureas)	0.15	0.301	7.566	[45]
2.	Indinavir (non-cyclic ureas)	0.14	0.221	7.524	[45]
3.	Nelfinavir (non-cyclic ureas)	0.28	0.312	8.441	[45]
4.	141W94 (non-cyclic ureas)	0.11	0.477	8.527	[45]
5.	Ritonavir (non-cyclic ureas)	0.17	0.213	7.815	[45]
6.	8 (cyclic ureas)	0.014	0.234	8.461	[46]
7.	42 (cyclic ureas)	0.016	0.204	8.864	[46]
8.	5 (cyclic ureas)	0.016	0.253	9.018	[47]
9.	SD 146 (cyclic ureas)	0.024	0.749	8.449	[47]
10.	1(cyclic ureas)	0.018	0.474	8.302	[48]
11.	7 (cyclic ureas)	0.018	0.122	8.994	[48]
12.	5b (cyclic ureas)	0.016	0.329	8.846	[49]
13.	XN 975 (cyclic ureas)	0.027	0.231	8.597	[49]
14.	15 (cyclic ureas)	0.012	0.271	9.124	[50]
15.	13 (cyclic ureas)	0.016	0.499	8.432	[50]

### Pharmacophore Description

Since, we have used two analogous nucleus i.e. cyclic cyanoguanides (15 compounds) and cyclic urea (18 compounds) bearing various substitutions at P2/P2′ groups, a thorough analysis of fitting of these molecules into hypothesis 1 revealed quite interesting results. Pharmacophore model was visually inspected by fitting most active compounds from both series, i.e. cyclic urea series **(9r, 9s)** as well as from cyclic cyanoguanides series **(8r)** in the training set on each generated model to investigate recurrent features.

**Figure 7:**
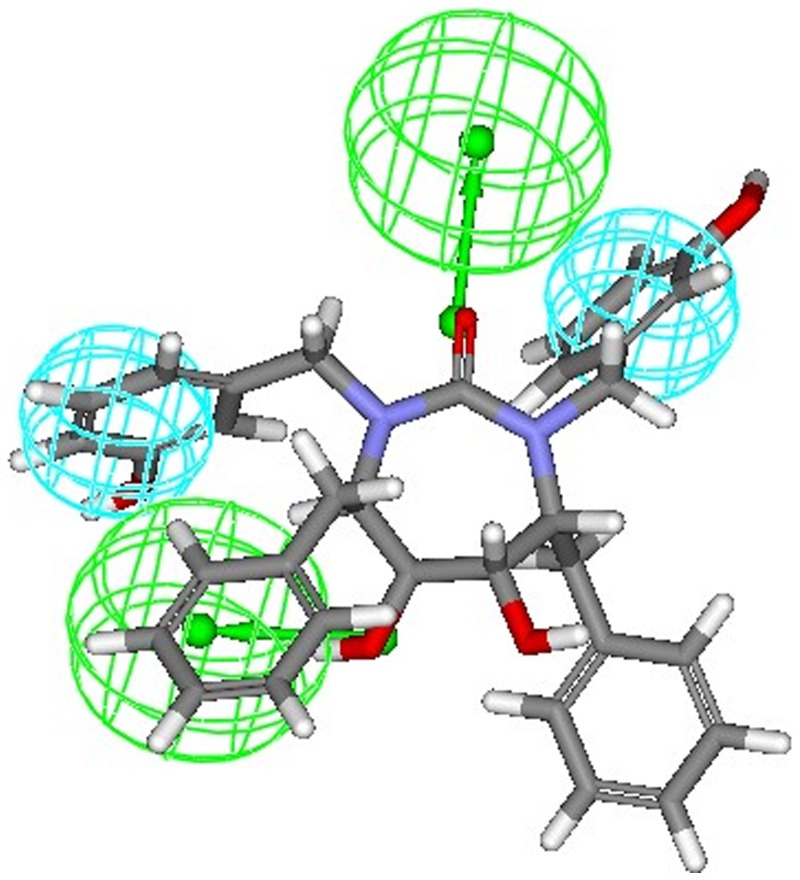
Mapping of most active compound 9r (cyclic urea derivative) onto the generated pharmacophore model (hypothesis 1).

**Figure 8:**
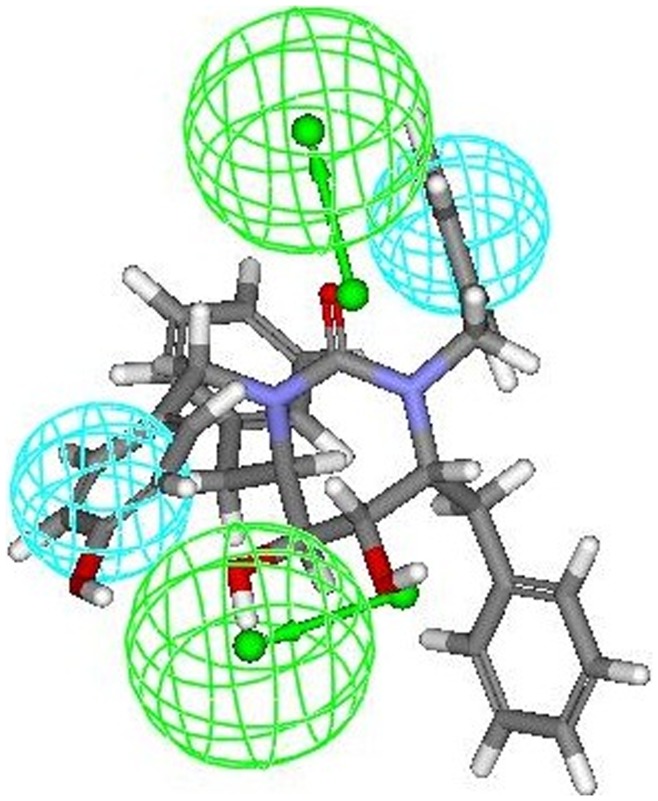
Mapping of another most active compound 9s (cyclic urea derivative) onto the generated pharmacophore model (hypothesis 1).

The most active compounds **9r and 9s** (from cyclic urea series) mapped perfectly well to all the four features of hypothesis 1. Compound **9r** (one of the most active compound) mapped with both the hydrophobic (HY 1 and HY 2) features of hypothesis 1 at the two benzene rings of 3-hydroxybenzyl groups at P2/P2′ positions. One of the two hydrogen bond acceptor lipid (HBA 1) feature was occupied by oxygen of the cyclic urea carbonyl group. Second hydrogen-bond acceptor lipid feature (HBA 2) was mapped onto one of the two symmetrical hydroxyl groups attached on the cyclic urea ring (or cyclic guanidine ring as applicable) (Fig. 7). This fact is also supported from the findings reported earlier that oxygen of the cyclic urea carbonyl group act as a hydrogen bond acceptor for backbone amides of flap residues Ile50/Ile50′ and hydroxyl groups attached on the cyclic urea ring behaves as hydrogen bond acceptor for carboxylate group of Asp25/Asp25′ (active site of HIV-1 protease is shared by both aspartyl subunits) [24], [40]. Similar trend of alignment of all the four features was also observed when another most active compound **9s** belonging to cyclic urea series was mapped into the pharmacophore derived from hypothesis 1 (Fig. 8). The two benzene rings of 4-hydroxybenzyl groups at P2/P2′ positions were exactly aligned towards both the two hydrophobic (HY 1 and HY 2) features of hypothesis 1. Oxygen of the cyclic urea carbonyl group occupied the first hydrogen bond acceptor lipid (HBA 1) feature and second hydrogen-bond acceptor lipid feature (HBA 2) of the selected pharmacophore was mapped onto one of the two symmetrical hydroxyl groups attached on the cyclic urea ring.

Out of 18 molecules from cyclic urea derivatives in training set, only two compounds namely **9a** and **9m** exhibited three features fit, rest all 16 compounds showed a perfect four feature fit proving the accuracy of the developed pharmacophore model for cyclic urea derivatives (Table 4).

**Figure 9:**
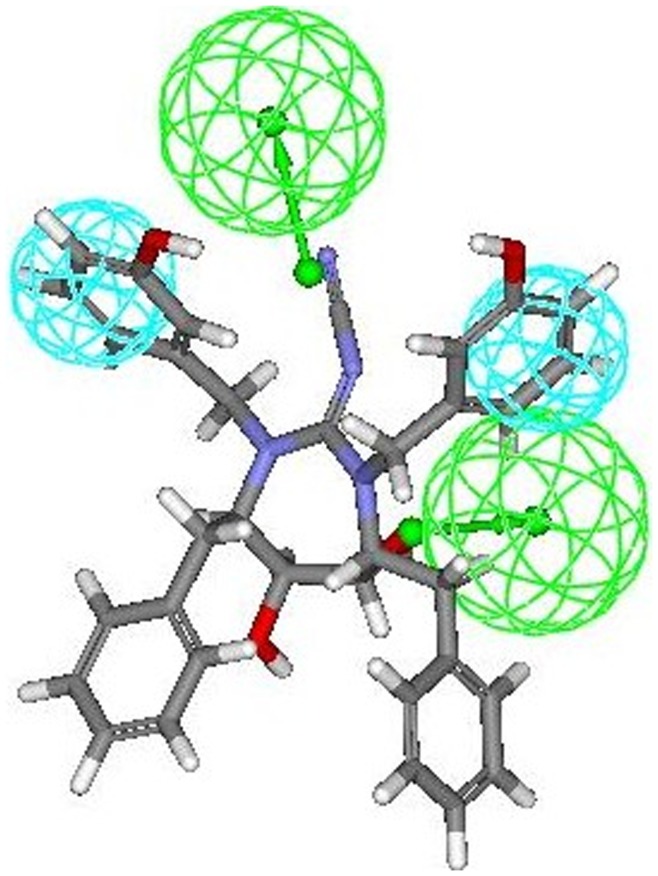
Mapping of compound 8r (cyclic cyanoguanidine derivative) onto the generated pharmacophore model (hypothesis 1).

Comparable mapping fashion was spotted out when **8r** (most active compound from cyclic cyanoguanides series) was mapped onto the developed pharmacophore. Four feature mapping was observed in which two hydrophobic (HY 1 and HY 2) features and second hydrogen-bond acceptor lipid feature (HBA 2) were associated at same position as that of **9r** (most active compound from cyclic urea series) i.e. at the two benzene rings of 3-hydroxybenzyl groups at P2/P2′ positions and one of the two symmetrical hydroxyl groups attached on the cyclic cyanoguanide ring at respectively. Another hydrogen bond acceptor lipid (HBA 1) feature of the hypothesis 1 was aligned towards exocyclic guanidine nitrogen of cyanoguanide ring (Fig. 9).

**Figure 10:**
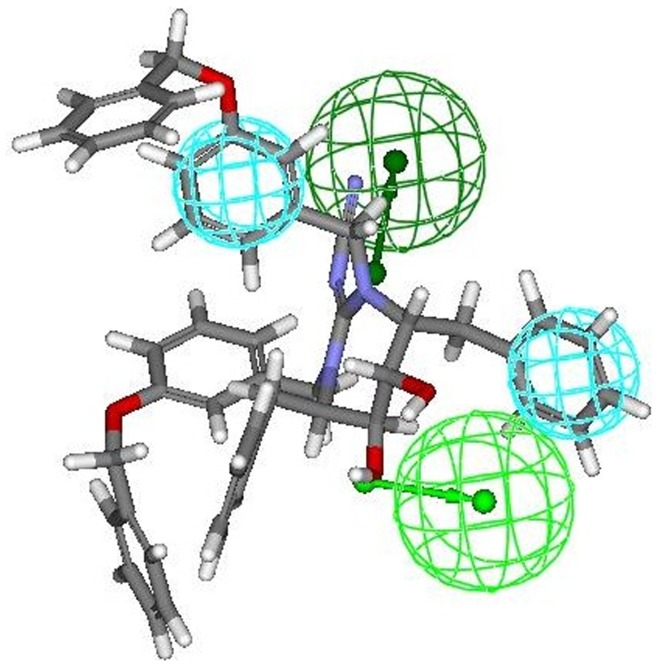
Mapping of least active compound 8t (cyclic cyanoguanidine derivative) onto the generated pharmacophore model (hypothesis 1).

**Figure 11:**
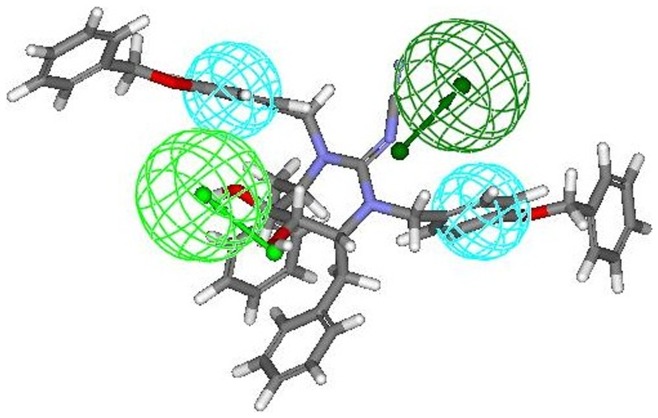
Mapping of another least active compound 8u (cyclic cyanoguanidine derivative) onto the generated pharmacophore model (hypothesis 1).

The mapping of pharmacophore obtained from hypothesis 1 onto inactive compounds assisted in clarifying the possible structural reasons for their inactivity which was demonstrated experimentally by these compounds, since the best “function mapping” covered three of the pharmacophoric features out of four features. Mapping of two least active molecules from training set namely **8t** and **8u** (belonging to cyclic cyanoguanides series) exhibited three feature mapping (Fig. 10 and 11 respectively), because the first hydrogen bond acceptor lipid (HBA 1) feature was missing due to orientation of exocyclic guanidine nitrogen in three dimensional space, which made it impossible to map onto the HBA 1 feature of the pharmacophore thus rendering them inactive. The same trend that HBA 1 feature could not effectively map exocyclic guanidine nitrogen was also seen with most of the candidates from cyanoguanides series. Therefore, we may draw a conclusion that oxygen of the cyclic urea carbonyl group will act as better hydrogen bond acceptor for backbone amides of flap residues Ile50/Ile50′ than exocyclic guanidine nitrogen, which is also supported by the earlier reports [24].

**Figure 12:**
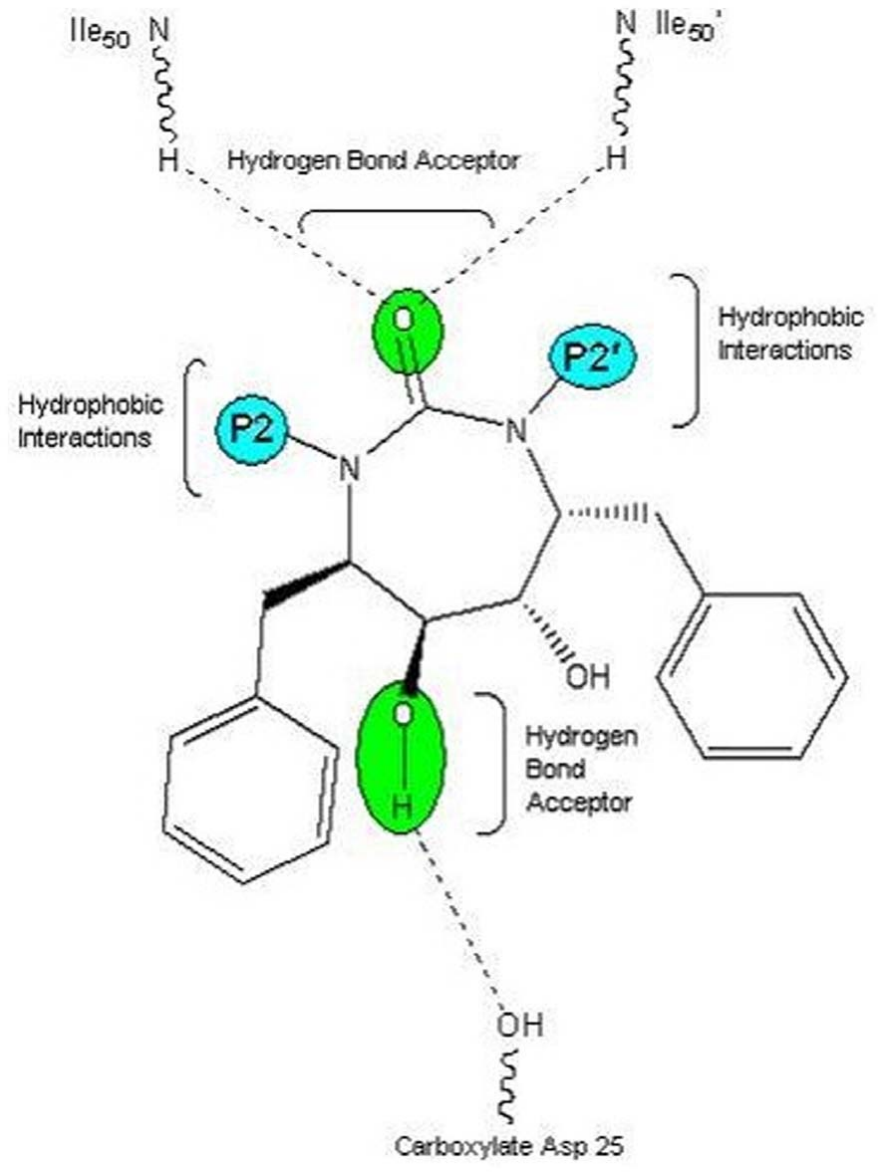
Proposed model for the interaction of symmetrical P2/P2′ cyclic urea with developed pharmacophore.

A proposed model for the interaction of symmetrical P2/P2′ cyclic urea with developed pharmacophore is shown in Fig. 12. From the figure, it is evident that while designing newer HIV-1 protease ligands, one must emphasize upon symmetrical cyclic urea derivatives (as oxygen of the cyclic urea carbonyl group act as hydrogen bond acceptor for backbone amides of flap residues Ile50/Ile50′) over cyclic cyanoguanides and also substitute the cyclic urea ring with lipophilic groups at P2/P2′ positions as evident from two hydrophobic (HY 1 and HY 2) features which were mapped accurately at P2/P2′ positions in all the candidates of data set. This observation is also augmented by report on X-Ray study performed by Bone et al. According to their findings, desirable features in an HIV-1 protease inhibitor would include hydrophobic substituents to project into the specific pockets of the enzyme and hydrogen-bond acceptor to interact with the carboxylate oxygens of both Asp 25 (active site is shared by both aspartyl subunits), which projects up from the floor of the active site from each subunit [33].

Lam et al. also enlightened the most important advantage of cyclic urea derivatives that the three-dimensional structure of the HIV PR complexes with other acyclic inhibitors revealed a unique structural water molecule which connects the inhibitor to the flap through hydrogen bonding interactions. The cyclic urea classes of inhibitors were able to displace this unique structural water molecule. A fundamental feature of these inhibitors is the cyclic urea carbonyl oxygen that mimics the hydrogen-bonding features of a key structural water molecule [41].

Recently, Sivan and Manga also emphasized on the importance of hydrogen bond interactions with the active site amino acids, carboxylate of Asp25 and amine of Ile50 [42]. Another study based on molecular dynamics simulation and binding free energy decomposition [43], suggested that the residues that make significant contributions to the binding are all hydrophobic amino acids.

### Structure Based 3D Pharmacophore Generation

#### Pharmacophore description and its comparison with pharmacophore obtained from ligand-Based study

**Figure 13:**
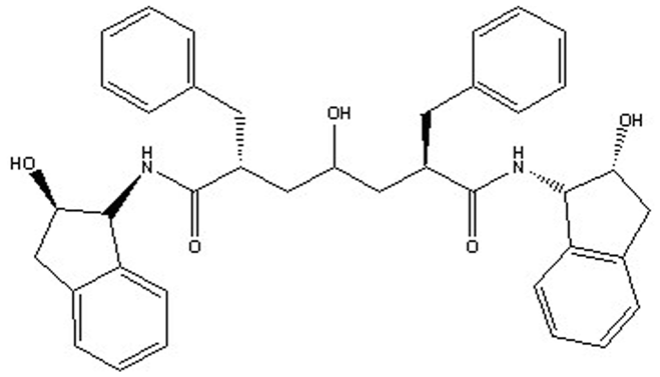
Structure of the ligand L-700,417 (*N,N-bis(2-hydroxy-1-indanyl)-2,6-diphenylmethyl-4- hydroxy-1,7-heptandiamide*).

**Figure 14:**
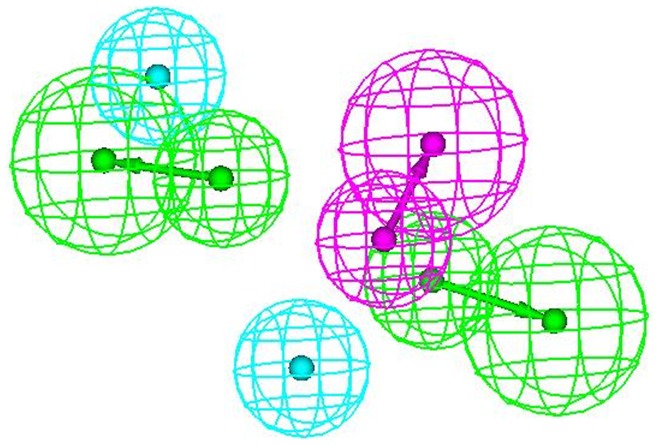
Pharmacophoric features retrieved through structure-based strategy.

An attractive application of receptor-based pharmacophore model is to discover interaction spots so as to guide the improvement of binding affinity and/or maximizing selectivity. The three-dimensional structure of HIV-1 protease enzyme complexed with inhibitor *L-700,417* (Fig. 13) was exploited to develop a pharmacophore model. Active site of HIV-1 protease was identified and highlighted by sphere of 9.0 Å. The pharmacophore generated from 3D structure of protease enzyme contained five features: one hydrogen bond donor (HBD) and two hydrogen bond acceptors (HBA) and two hydrophobic groups (HY) (Fig. 14). Green, blue and magenta colors are represented by hydrogen bond acceptor, hydrophobic and hydrogen bond donors features respectively.

The comparison of pharmacophoric features obtained from structure-based and ligand-based study revealed that both the pharmacophores have four common points i.e. two hydrogen bond acceptors (HBA) and two hydrophobic groups (HY). The pharmacophore obtained from structure-based study exhibited one additional feature i.e. hydrogen bond donors (HBD). This observation revealed that along with HBA and HY features, HBD feature can also contribute an additional interaction site at HIV-1 protease. All the 47 compounds of the compound library were mapped onto the generated structure-based pharmacophore. One of the interesting outcome of the study was that out of different conformations of 47 compounds, 351 (various conformations) hits were obtained and 41 hits exhibited a five-feature mapping and rest all showed a four-feature interaction. These hits presented the chemical features and the shape suggested by the structure-based pharmacophore model.

**Figure 15:**
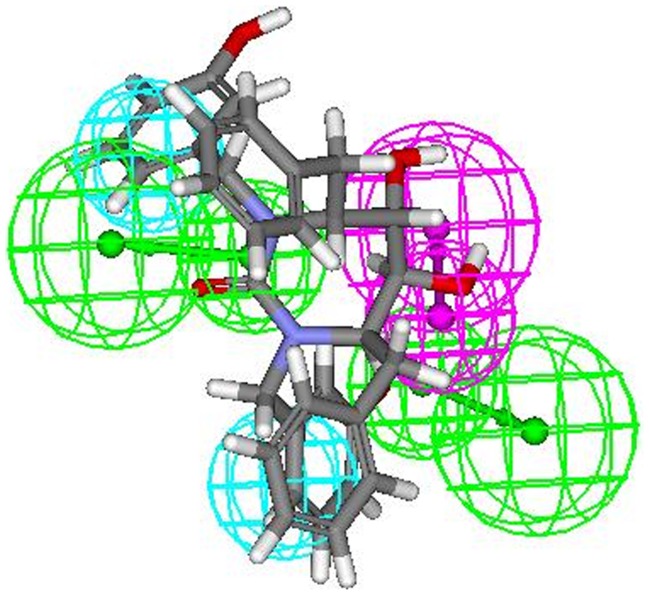
Pharmacophoric interaction of most active compound 9s onto the pharmacophore obtained from structure-based approach.

**Figure 16:**
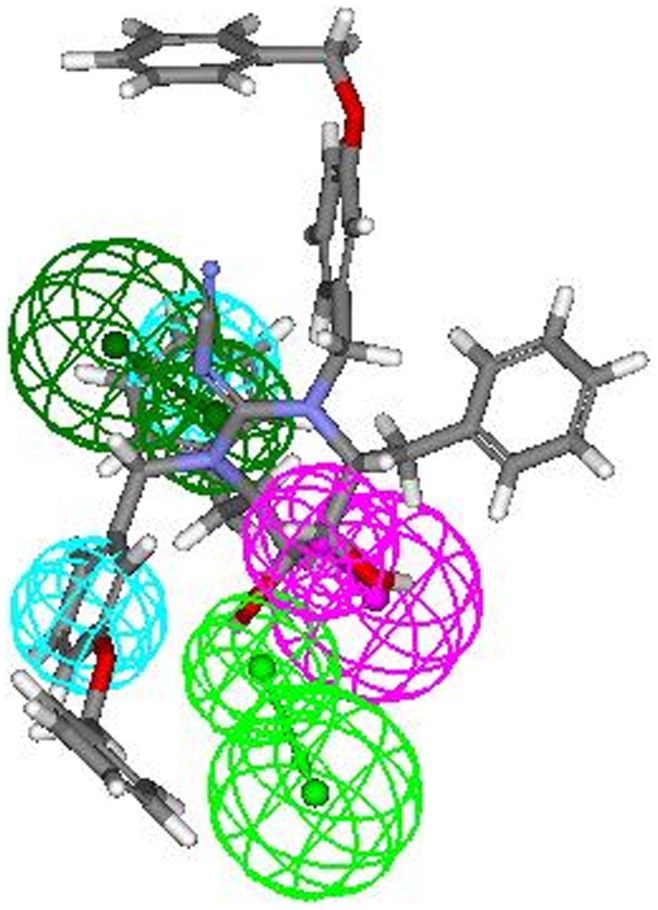
Pharmacophoric interaction of least active compound 8t onto the pharmacophore obtained from structure-based approach.

Most active compounds **9r** and **9s** displayed a perfect five-feature fit. The thorough analysis of pharmacophoric interaction of two most active compounds **9r** and **9s** (Fig. 15) revealed that the oxygen of the cyclic urea carbonyl group involved in hydrogen bonding (HBA 1) which seems to be essential for activity. Also, hydroxyl group present at P2/P2′ positions of ring acts as hydrogen bond acceptor (HBA 2) for amino acids present at the active site. The benzene ring at P2/P2′ is essentially involved in hydrophobic interaction with surrounding hydrophobic amino acids (HY1 and HY2). These all above observations exactly matched with the positions of all 4 (2 HBA and 2 HY) features obtained through ligand-based analysis when mapped onto most active compounds **9r** and **9s** confirming the accuracy of the HypoGen pharmacophore. Another feature i.e. hydrogen bond donor (HBD) which was additionally retrieved through receptor-based approach was mapped onto the hydrogen of second hydroxyl group at the ring. Mapping fashion of least active compound **8t** onto the structure-based pharmacophore was also analyzed, which exhibited a four-feature fit in which hydrogen bond acceptor (HBA 1) was missing due to absence of cyclic urea carbonyl group (cyclic cyanoguanidine moiety) and hence resulted in least biological activity (Fig. 16). Interestingly, comparison of pharmacophoric interactions of both the pharmacophores (obtained from ligand as well as structure based study) display common binding mode and indicates the significance of hydrogen bond acceptor, donor and hydrophobic functionalities in defining the activities of compounds.

It is also interesting to note that the seventeen different conformations of the compound **9s** were obtained as hits, out of seventeen conformations sixteen mapped to four features of the input pharmacophore whereas one mapped to five features, i.e. two hydrogen bond acceptors, two hydrophobes and one hydrogen bond donor (Fig. 15). It seems that one out of seventeen different conformers is able to adopt a orientation which can interact with all five pharmacophoric features at HIV-1 protease binding pocket due to conformational adjustment. Hence, the model developed herein also highlights the importance of bioactive conformation in eliciting the biological response.

**Figure 17:**
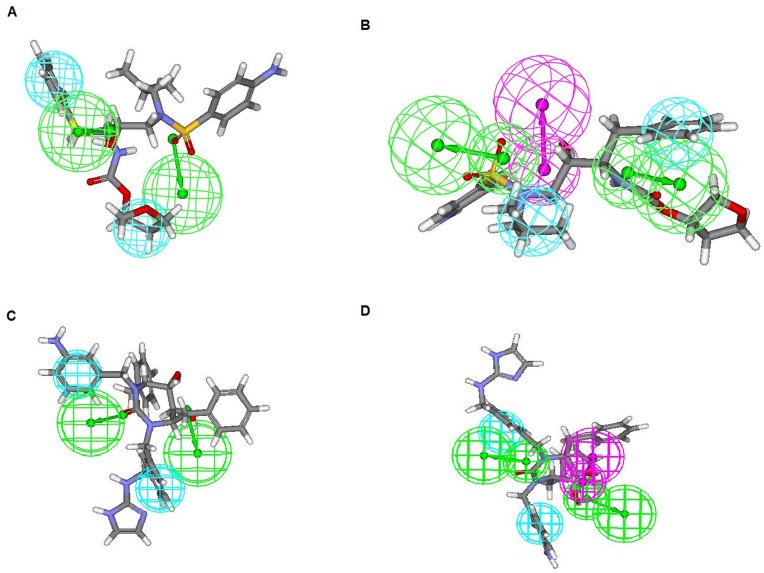
Mapping of the most active compounds from external validation set. Most active non-cyclic urea derivative (141W94) mapped onto: (A) hypothesis 1(four feature mapping) and (B) structure based pharmacophore (five feature mapping). Most active cyclic urea derivative (15) mapped onto: (C) hypothesis 1 (four feature mapping) and (D) structure based pharmacophore (five feature mapping).

Also, 15 external test set molecules which were used to validate the pharmacophore developed from ligand-based methodology were also used as a screening validation dataset on the five-feature structure-based pharmacophore. All the 15 external test set molecules exhibited good estimated activities and fit values (shown in Table 6) explaining the accuracy of our developed pharmacophore. The most active compounds of both non-cyclic and cyclic urea derivatives showed the best fit values (Fig. 17 and Table 6).

### Database Mining

**Figure 18:**
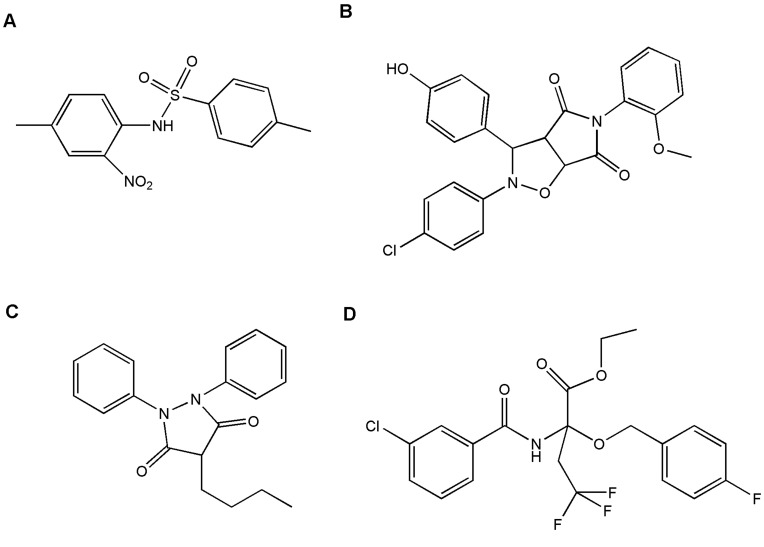
Chemical structures of hits obtained from Maybridge database. (A) BTB01434, (B) BTB14348, (C) BTB12395 and (D) BTB13591.

**Figure 19:**
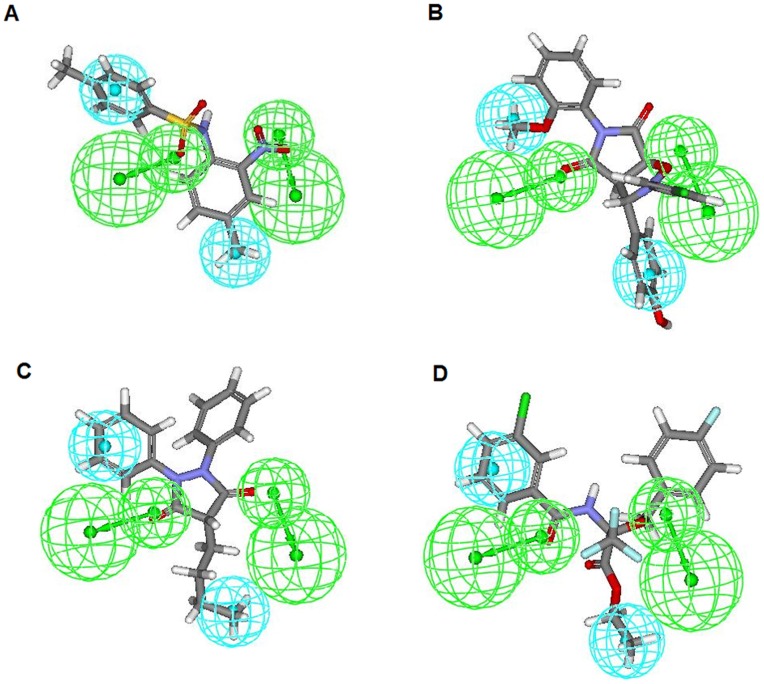
Pharmacophore mapping of Maybridge hits onto hypothesis 1. (A) BTB01434, (B) BTB14348, (C) BTB12395 and (D) BTB13591.

The validated pharmacophore obtained from HypoGen analysis i.e. hypothesis 1 was used to screen molecules with similar features from the Maybridge and NCI database to find other structural motifs that fulfill the functional and spatial constraints of the model. This method constitutes a powerful way for quickly finding new potential lead compounds in a medicinal chemistry project. As a result of this search, 399 lead compounds were obtained from the first 3D query and their activities were estimated, out of which 4 candidates namely BTB01434, BTB14348, BTB12395 and BTB13591 (Fig. 18) turned out as potential ligands exhibiting a good perfect four feature fit (fit value being 8.568, 8.141, 8.005 and 7.944 respectively) from which one lead BTB01434 exhibited quite good predictive activity of 0.798 nM (Table 7). The four features of the above pharmacophore were mapped onto the best estimated compound BTB01434 (Fig. 19A) in the following manner: HBA 1 was occupied by carbonyl oxygen from sulfonamide moiety and HBA 2 mapped well onto oxygen belonging to nitro group. While first hydrophobic (HY 1) feature mapped onto benzene ring directly attached to sulfonamide group and another hydrophobic (HY 2) feature took its position towards the 4-methyl group attached with second benzene ring. Pharmacophore mapping of all four Maybridge hits onto hypothesis 1 is shown in Fig. 19.

**Table 7:** Hits retrieved through database screening along with their estimated activity and Lipinski’s parameters.

Name (Product Namefrom Maybrige)	Estimated Ki (nM)values	Fit Value	cLogP	Molecular weight	H bonds donor	H Bonds acceptor
BTB 01434	0.79	8.568	2.63	306.33	1	6
BTB 14348	1.03	8.141	4.01	450.87	1	7
BTB 12395	1.07	8.005	2.97	308.38	0	4
BTB 13591	2.36	7.944	4.65	433.78	1	5

### Validation of Database Compounds

In order to validate the pharmacophoric pattern of above mentioned four database compounds, their conformations were generated and mapped onto the pharmacophore derived from structure-based strategy. Out of different conformations of four compounds, 19 (various conformations) hits were obtained and three hits (each from BTB01434, BTB14348, and BTB13591) exhibited a perfect five-feature mapping and rest all showed a four-feature interaction. Analysis of the best five feature hit exhibited by highest estimated compound BTB01434, revealed that two HBA and two HY features were mapped exactly on the same groups as that of mapping obtained onto the pharmacophore obtained from HypoGen study. An additional feature i.e. hydrogen bond donor (HBD) which was additionally retrieved through receptor-based approach was mapped onto the –NH group of suphonamide moiety. This similar pattern of accurate mapping (common mapping fashion) was seen, when rest of three database compounds were mapped onto the structure-based pharmacophore, hence proving the precision of our predicted database compounds. Therefore, on the basis of above validation of these database compounds through mapping onto structure-based pharmacophore, we conclude that these database compounds would ensure good Ki values if experimentally synthesized and pharmacologically evaluated for HIV-1 enzyme inhibitory activity.

Lipinski’s “rule of five” is a heuristic approach for predicting drug-likeness stating that molecules having molecular weight >500, log P>5, hydrogen bond donors >5 and hydrogen bond acceptors >10 have poor absorption or permeation [44]. The parameters included in Lipinski’s rule of 5, were calculated for the above four molecules retrieved from database search and are summarized in Table 7 along with their fit value and estimated activity. The results clearly indicate that there is no violation to Lipinski’s rule and it is highly likely that all the designed compounds will also have favorable pharmacokinetics profile.

### Conclusions

In this study, we described the development of highly selective pharmacophore models for inhibitors of HIV-1 protease. The generated pharmacophore reflects the binding mode and the important interactions of the ligands with certain amino acids in the active site of HIV-1 protease enzyme. In our ligand-oriented study, efforts were made to take multiple contributions of ligand features to build a quantitative pharmacophore models from a training set of 33 HIV-1 protease inhibitor analogs. The best pharmacophore consisted of four pharmacophore features, including two hydrogen bond acceptor and two hydrophobic features, having a correlation coefficient of 0.90. Besides, this hypothesis was further validated by an external test of 15 compounds. The type and spatial location of the chemical feature agree perfectly with the pattern of enzyme inhibitor interactions identified from crystallography. In our structure-oriented study, another 3D pharmacophore model from HIV-1 protease enzyme was developed and was used to screen compound library comprising of for HIV-1 protease inhibitors as validation step. The interaction shown by the compounds provides the insight into the mechanism involved in the ligand-enzyme interaction. The model developed herein also highlights the importance of bioactive conformation in eliciting the biological response. As a result of database mining four structurally diverse protease inhibitors were identified.
